# Editorial: Post-translational modifications of proteins in cancer immunity and immunotherapy

**DOI:** 10.3389/fimmu.2022.1006145

**Published:** 2022-08-12

**Authors:** Xiangpeng Dai, Jinfang Zhang, Brian J. North, Jianping Guo

**Affiliations:** ^1^ Key Laboratory of Organ Regeneration and Transplantation of Ministry of Education, First Hospital, Jilin University, Changchun, China; ^2^ National-Local Joint Engineering Laboratory of Animal Models for Human Disease, First Hospital, Jilin University, Changchun, China; ^3^ Frontier Science Center for Immunology and Metabolism, Medical Research Institute, School of Medicine, Wuhan University, Wuhan, China; ^4^ Biomedical Sciences Department, Creighton University School of Medicine, Omaha, NE, United States; ^5^ Institute of Precision Medicine, the First Affiliated Hospital, Sun Yat-sen University, Guangzhou, China

**Keywords:** post-translational modifications, PD-1/PD-L1, tumor microenvironments, immune checkpoint, cancer immunotherapy

In 2018, Tasuku Honjo and James Allison shared the Noble Prize in Physiology and Medicine for their discoveries in cancer immunology and immune checkpoints (ICs). Over the past few decades, with the investigations of ICs, cancer immune therapies have been extensively studied and have progressed all the way to clinical usage for a variety of cancer indications (1). However, limited response (~25% of all cancer types) often due to limited immune response to the tumor, and acquired resistance, markedly restrict clinical application of immune checkpoint inhibitors (ICIs) (1, 2). Therefore, elucidating the mechanisms of tumor immune responses and optimizing combinatorial strategies to promote a more robust immune response to a tumor have gained more attention. Recent studies have pointed to protein post-translational modifications (PTMs) as playing critical roles, either directly or indirectly, in influencing immunotherapeutic efficacy by modulating ICs and/or shaping the tumor immune microenvironments (TIMEs) (3, 4). Conceivably, continued focus on these areas of regulation will allow for further development of promising strategies to increase efficacy of cancer immunotherapies (5). Thus, we have assembled this collection of articles to provide an overview as well as original findings for PTMs in cancer immunity and immunotherapy.

## PTMs in modulating ICs

**Figure 1:**
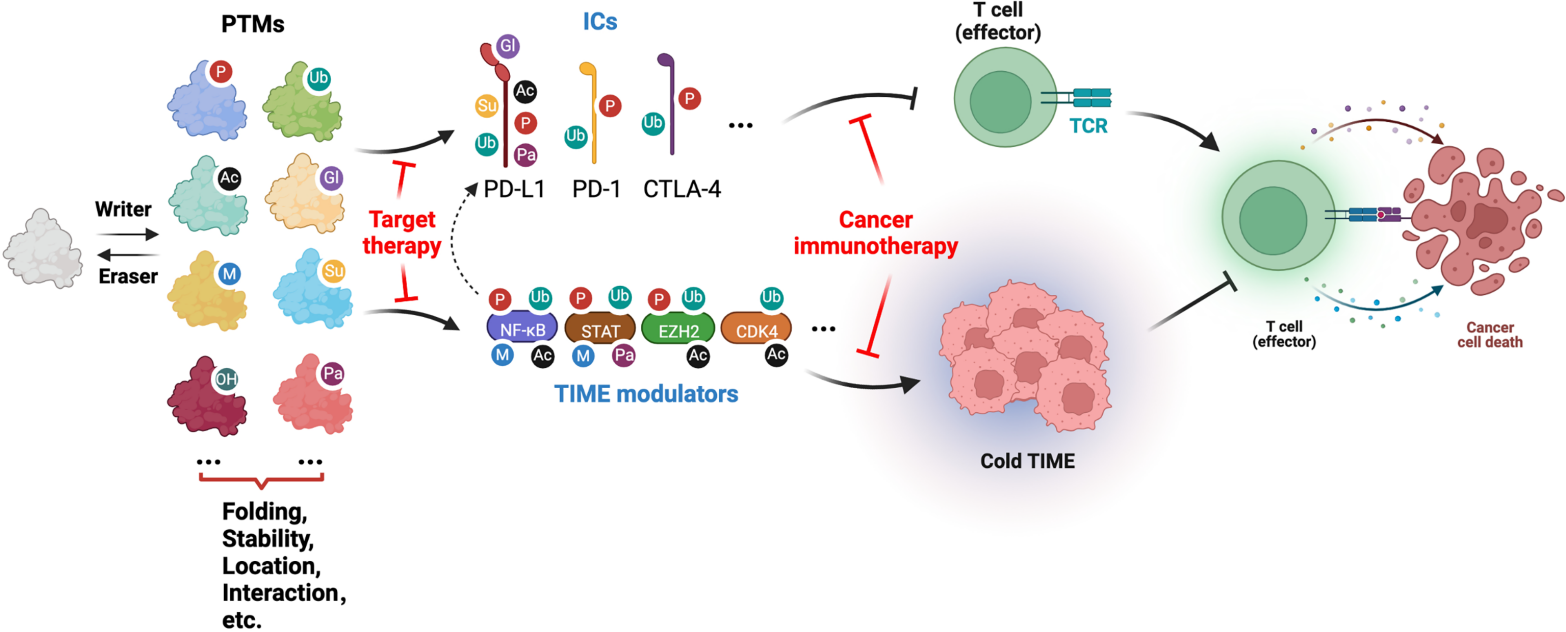
Illustration of PTMs involved in cancer immune and immunotherapy by modifying ICs and TIME modulators (generated with Biorender.com).

PTMs are well-established covalent modifications to amino acid side chains of proteins that are catalyzed by a growing list of enzymes. Of note, a majority of PTMs are reversible and dynamically regulated, and play a significant role in expanding proteome diversity and regulating protein functions in various biological or pathological processes, including immune diseases and cancers (**Figure 1**). To date, while only a handful of PTMs have been extensively studied, more than 450 PTMs have been identified, that regulate many aspects of protein function by governing their folding, stability, localization, catalytic activity, and binding proteins. Recently, PTMs modulating ICs and TIMEs are being explored for their role in regulating cancer immunity and immunotherapies (**Figure 1**). Notably, phosphorylation, a well-established and studied PTM, function in regulating ICs have been recently reviewed, where PD-1/PD-L1 and other ICs including CTLA-4, Tim3 and TIGIT, all undergo phosphorylation-mediated regulation, providing potential novel strategies to target kinases, in particular receptor tyrosine kinases (RTKs), in combination with ICIs for cancer immunotherapy (5, 6).

Besides phosphorylation, ubiquitination is another ubiquitous PTM, and mainly functions in governing protein degradation. E3 ubiquitin ligases, which catalyze the addition of ubiquitin to substrate proteins, determine the ubiquitin types and substrate specificity, thus their functions in regulating degradation of IC factors are becoming more appreciated (7), and provide potential targets for cancer immunotherapies. While the role of conjugating E2 enzymes, which serve as mediators of E3 ligases, in cancer immunity remains elusive. However, in this collection, the E2 enzyme UBE2L3 is described in more detail including its link with cancer immunity has been explored (Zhang et al.). Briefly, UBE2L3 exhibits abnormal expression in a variety of disorders, including tumor and immune diseases, potentially through modifying NF-κB, DNA repair and autophagy pathways, thus providing a potential target for cancer immunotherapy. Moreover, further studies exploring whether and how other E2 enzymes play potential roles in cancer immunity and immunotherapies are warranted.

Lysine acts as one of the most common modified amino acids. Besides undergoing ubiquitination, it is also subjected to acetylation, methylation, SUMOylation, and likely many other PTMs. Among these modifications, acetylation has been broadly characterized to function in epigenetic regulation (8). In this collection, a role for acetylation and its modifying enzymes in regulating ICs directly (acetylation of PD-L1) or indirectly (acetylation of transcriptional factors or histones to regulate ICs expression) have been comprehensively overviewed (Ding et al.). Meanwhile, modulators of the writers and erasers of acetylation, such as histone acetyltransferase (HATs) and histone deacetylases (HDACs), have undergone extensive anti-cancer therapy development and have been studied for their potential as combination agents for cancer immunotherapy. Nihira and Miki provide a review in this collection highlighting acetylation in regulating ICs. Besides its canonical functions as a plasma membrane bound ligand for PD-1, PD-L1 also plays an important role in the nucleus controlled by its acetylation (7). These nuclear functions of PD-L1 include modulating transcriptional regulation, cell death, regulation of mRNA stability and restricting the interferon (IFN) and mTOR signaling pathways, to contribute tumorigenesis and TIME modulation.

## PTMs in shaping TIMEs

With the in-depth study and clinical development of ICIs-associated cancer immunotherapies, crucial roles of TIME status have come to the forefront (3, 4). A major driver of ICI efficacy is the presence of a potent immune response developed towards the tumor. Identifying approaches to alter TIMEs from desert to inflamed (also as from cold to hot) are promising strategies to enhance the efficacy of cancer immunotherapies (4). Although prior reviews in the literature have summarized that PTMs such as phosphorylation and acetylation are involved in TIME modulation (6), an overview of arginine methylation in cancer immunotherapy has yet to be discussed. A comprehensive review provided by Dai et al. in this collection reveals that, besides their epigenetic modification functions in transcription and RNA processing, arginine methylation is involved in shaping TIME and cancer immunotherapy, although this PTM has yet to be identified to directly modify ICs. The function of protein arginine methyltransferases (PRMTs), the writers of arginine methylation, in shaping TIMEs has been demonstrated at multiple levels. Among these, PRMTs promote tumor antigen presentation, regulate the secretion of chemokines, overcome inhibitory networks as well as modulate signaling pathways in innate and adaptive immune responses during tumorigenesis by targeting a wide range of proteins for arginine methylation. Thus, targeting PRMTs in combination with ICIs could provide a novel combination to increase ICI efficacy in patients.

To help define the status of TIMEs, clinical biomarkers for immune response within tumors have been extensively explored, such as through bioinformatic analyses from tumor tissue-based single cell sequencing and immunohistochemistry (9). In this collection, two original studies have provided further examples of using PTMs to monitor TIMEs in patients. Xia et al. have defined SUMOylation patterns that may be used to predict immune response for bladder cancers, thus providing important information to guide clinical cancer immune therapy strategies in bladder cancer. In addition, using a bioinformatics approach, Tu et al. evaluated protein disulfide-isomerase A3 (PDIA3) as a biomarker for immune response in urological tumors as well as melanoma, which they further expanded to a more extensive list of cancer types. However, due to a lack of evidence as to whether these PTMs directly modify ICs, their direct roles in acting as biomarkers associated with TIME status to predict the potential therapeutic response to immunotherapies require further investigation. These recent findings suggest that further understanding PTMs and their modifying enzymes as biomarkers for TIMEs may provide important information to guide cancer treatment strategies.

## Conclusion

This Research Topic provides an overview of the interrelationship between PTMs in cancer immune response and immunotherapy by regulating ICs and their modulators in shaping TIMEs (**Figure 1**). With the development of *in vivo* CRISPR-based screening approaches, high resolution *in vivo* mass spectrometry analyses, and next-generation single cell sequencing technologies, there is no doubt that additional PTMs in ICs and TIME modulators will be elucidated. These studies will not only help to refine our understanding of PTMs in modulating cancer immunity, but also provide potential targets for combination strategies to increase therapeutic efficacy of ICIs for cancer treatment.

## Author contributions

XD, JZ, BN and JG edited the Research Topic and wrote this editorial. All authors approved the submitted version of this editorial.

## Acknowledgments

The authors sincerely apologize to all those colleagues whose important work was not cited in the paper owing to space limitations.

## Conflict of interest

The authors declare that the research was conducted in the absence of any commercial or financial relationships that could be construed as a potential conflict of interest.

## Publisher’s note

All claims expressed in this article are solely those of the authors and do not necessarily represent those of their affiliated organizations, or those of the publisher, the editors and the reviewers. Any product that may be evaluated in this article, or claim that may be made by its manufacturer, is not guaranteed or endorsed by the publisher.
